# Disclosing mental illness: a doctor's dilemma

**DOI:** 10.1192/bjb.2020.25

**Published:** 2020-12

**Authors:** Rebecca Lawrence

**Affiliations:** Ritson Clinic, Royal Edinburgh Hospital, UK

**Keywords:** Stigma and discrimination, education and training, supervision, ethics, physician health

## Abstract

There is increasing evidence that doctors have high levels of mental illness, and there are concerns that, for some, this may be exacerbated by their working environment. It can be difficult for doctors to disclose mental illness, either to senior or junior colleagues, and perhaps even harder to know what, if anything, to say to patients. Many doctors may be unsure of their position as regards disclosing to governing bodies; others may disclose widely on social media. I am a psychiatrist who also has a significant mental illness, and refer both to my personal experience and the literature to explore some of these issues.

I became a consultant psychiatrist 14 years ago. When I started life as a psychiatric trainee, some 9 years prior, I had already been advised that this was not a good idea, as it would probably be too stressful for me. You might wonder why, given that I had previously completed general practitioner (GP) training; but I had also had the misfortune to have been a psychiatric patient. Briefly, I became ill in my GP trainee year, with what was almost certainly a perinatal mood disorder. I was in and out of hospital over the following 2 years, and had several courses of electroconvulsive therapy (ECT), before finally recovering sufficiently to return to work.

This was one of the hardest times of my life. I had minimal help with this return, and just had to apply for locums, at which I failed, and then 6-month junior hospital posts. There was no phased return, and I did full resident on-call from the start. I was determined and driven – I had to be. Just your average doctor, really, and with the usual tunnel vision. I regret this time, when I was a new mother, and recovering – it should have been gentler – but I felt that unless people saw me as tough, there was little hope of ever returning to work.

I was diagnosed with psychotic depression, and subsequently with bipolar disorder. After that first episode, I believed that I would never be ill again, until it became apparent, even to me, that this was a long-term condition, something that would probably never go away entirely. I have indeed had relapses, and my most recent encounter with ECT was this past year.

The high risk of anxiety, depression and suicide in doctors, particularly anaesthetists, general practitioners and psychiatrists, has been highlighted by Clare Gerada, and a link made between fear of exposure and suicide.^1^ A recent survey by the British Medical Association (BMA) has emphasised the concerning levels of mental health problems in doctors and students, and how their working environment may have contributed to these.^2^ Some of the reasons for these difficulties have been examined in Caroline Elton's book, *Also Human: The Inner Lives of Doctors*,^3^ which explores the stresses unique to medicine, the way doctors can be treated and what happens when they can no longer carry on.

Over the years I have chosen at times to disclose my illness, and at times to keep silent – probably more often somewhere in between, leaving out the nasty bits. My first really public disclosure was a *BMJ* article in 2012,^4^ but I had always been fairly open with colleagues. Interestingly, doctors do not always behave as they think they will, with younger doctors and doctors in training less likely to disclose mental illness.^5^

How one decides when to disclose, and how much should be said, often depends on the context. Initially I felt that I was the only doctor to whom this had happened, and only when a kind supervisor gave me an article by the late Belinda Brewer^6^ did it occur to me that this was unlikely. That brief article affected me profoundly, and I have found reading personal accounts hugely helpful, as both a patient and doctor. *Doctors as Patients*, edited by the late Petre Jones,^7^ contains many different accounts, as well as guidance and advice, and is a book I have come back to when unwell. It is sobering to reflect on the loss of both of these doctors, and I want to acknowledge their courage.

Rees et al have acquired a considerable amount of qualitative data from interviewing doctors, and have commented on the importance of the reaction to the first disclosure of mental illness, whoever this is made to.^8^ This has obvious implications for the training and support of those who actually support junior doctors and medical students.

## Disclosing to patients

Disclosure of one's own illness to patients is a thorny area, and I do not think I have ever done it, although I have at times been tempted. But I have always thought this will make it more about me than them. It will close down the channels of communication, as my feelings become theirs. I work in a chronic pain clinic, and do not share my sciatica woes either. However sore I am, I cannot actually feel their pain.

De Vos et al discuss the use of disclosure by therapists in recovery, and make the important point that this must be done thoughtfully, without specific details of symptoms, and with support.^9^ There are growing numbers of blogs about the experience of mental illness, including those by doctors and psychiatrists. It is clear upon reading them that there is no one answer, implying that a great deal of consideration must be taken to ensure good patient care while treading sensitively. Howe specifically explores self-disclosure in psychiatrists, and concludes that a psychiatrist should only disclose what they are comfortable with, and what will benefit the patient.^10^

As a patient, I do not think that I would want my psychiatrist to disclose anything too personal to me. If they told me that they had been depressed, I think I would, in a peculiar way, feel out-manoeuvred, that there was no point in describing things further, as they already knew. Yet there can also be the odd sense that one's psychiatrist does not actually exist as a person themselves, leading to curiosity and sometimes alienation, which may be stronger when the patient is also a doctor.

As a doctor, my hope is that my experience will come through, that I will show rather than tell, and that this may influence my interactions with patients. I know that there is a school of thought that demonstrating one's own vulnerability may provide hope to a patient. Even then, I think the time to do so would be when the patient is better and less vulnerable themselves, as there is always a power imbalance in the patient–doctor relationship.

There is, of course, the reality that a patient may ask about your personal experience, particularly if you write or talk about it. I have always done this to some extent, but have expanded my efforts recently, both on Twitter and by blogging, and was interviewed on television earlier in 2019.^11^ It is perhaps easier for me as an established consultant, with supportive colleagues and managers, but my main reason for speaking out is that my children are older now, and understand more about my illness and why I would speak about it. As regards to patients, I would always tell them if they asked a direct question. I might say that the focus should be on them, and I might say that I would rather not discuss it in any detail, but I would not deny it.

## Disclosing to colleagues

Applying for training is both harder and easier now. I applied to several local training schemes, resulting in one outright rejection and two interviews. At both, I told them of my illness; one offered me a shorter contract in case I was not up to it, but the other just offered me the job. I was, and am, very glad that they knew of my illness from the beginning, and I experienced no stigma during my 2 years there, only support. But one would not now mention this at interview, which would, in any case, be done nationally. This lessens prejudice, but it also lessens your chance of discovering the right place to work, which I was fortunate to do. In other words, not being politically correct can, at times, be a bonus.

Throughout my training, I mostly worked with great consultants who knew my history, but I also tried to seek such people out whenever I could. There will always be those who, for whatever reason, are less able to support a trainee with difficulties, and my advice would be to avoid them whenever possible. The Royal College of Psychiatrists has resources for doctors and trainees returning to work,^12^ as does the Psychiatric Trainees Committee,^13^ which can be helpful both for those returning and those supporting them.

Perhaps I share too much, but I would advise trainees to definitely tell their educational supervisors, and usually their clinical supervisors, of any significant mental illness (definitely if not entirely well). My own problems had been rather public, being an in-patient in the local hospital, so I felt I had little option. You do not have to tell everyone with whom you work, any more than you have to tell them about other conditions, but sharing with your supervisors can make life smoother. Sometimes it is hard to do this face to face; I would then consider emailing what you want them to know, explaining that it can be difficult, and this can give both some time to reflect before speaking.

## Disclosing to governing bodies

The main body that governs doctors is the General Medical Council (GMC), and this can become very frightening when unwell. Unless a doctor is attempting to work against medical advice, the GMC should not become involved and the doctor should receive local support and treatment. This can include taking time off, but a reduction in hours or a phased return may also be recommended, and occupational health are well placed to help with this. There is no automatic need to inform the GMC unless there are legal issues, such as a drink driving charge, or other probity issues, when self-disclosure for health assessment should first be encouraged.^14^

Those doctors who are seen for health assessments face a number of possible outcomes, including not working for a period or working under supervision. I work as a health examiner and supervisor, and have seen how difficult this can be; there is undoubtedly much fear and stigma, but doctors often do very well, and many return to work. The GMC continues to work hard to dispel these fears as much as possible, and to provide more support, particularly following the concerns around increased suicide rates;^15^ but it is vital that doctors also receive support and validation from other sources, given that this can threaten the very meaning and identity of being a doctor.

When medical students apply to join the medical register, they too need to disclose any health conditions that may affect their fitness to practise.^16^ Medical schools have processes to manage students’ fitness to practise, and should be able to provide help and advice for those with pre-existing conditions.^17^

There are services available, such as the Psychiatrists’ Support Service,^18^ which provides anonymous telephone support to all grades of psychiatrist, and NHS Practitioner Health,^19^ which launched an immediate crisis text line this year, available to all doctors in England and Wales. Others include the BMA Doctor Support Service^20^ and the Doctors’ Support Network.^21^ DocHealth is a brief psychotherapy service available across the UK, staffed by medical consultant psychotherapists, and subsidised by the BMA and Royal Medical Benevolent Fund.^22^

## Disclosing being a doctor

It is extraordinary that saying one is a doctor can sometimes be harder than saying one has a mental illness. I was once part of a patient and carer group at the Royal College of Psychiatrists; it was a good experience, but I left because I did not really fit in, being a doctor as well as a patient. It is probably even harder being a doctor when an in-patient on a psychiatric ward, but this was not something that I ever broadcasted. I remember a patient shouting at me when, as a junior doctor, I visited a ward where I had previously been a patient: ‘You used to be one of us [patients], and now you're one of them [doctors] and how did that happen?’ At times I felt like a combination of a fake patient and an incompetent doctor.

## Disclosure to the wider community

More recently I have posted on Twitter, making it obvious I am both doctor and patient. The same things that garner support for a patient can enrage the online community when a doctor is involved. For me, one obvious example has been ECT, which I had this past year and have had previously. However, I recognise that although I am trained as a psychiatrist, my personal experiences are anecdotal and I try to present them as such, rather than generalising. I think this does take the heat out of things, although not always. I would, however, advise others not to post on Twitter when less well; there are many other gentler online forums that can provide helpful support. I have used the Doctors’ Support Network forum, which is completely anonymous, and a specific group for doctors with bipolar disorder.

I have started to speak and write more publicly about my illness,^11^ although I find that it is actually quite easy to hide behind a pen or a lens. I have also spoken directly to both trainees and retired psychiatrists, which is more daunting, but also more rewarding; and I hope to continue to tell my story to others, both to help them know that they are not alone and to highlight what support is out there.

## Receiving disclosures

Inevitably as a psychiatrist and trainer I have received disclosures from others regarding mental illness and other sensitive issues. I find this hard, and I think it is right to find it hard, not least because it has usually been very difficult for the trainee to raise. Acknowledge it, make no assumptions and ask how much the trainee wants to say. Ask them if they want to talk about it again, and think about what they have told you. Most importantly, remember that they are not you. Treat them normally, and do not make them feel that they have become ‘special’ or ‘different’. Help them, and guide them to find treatment if necessary, but do not be their doctor. Getting the balance can be very difficult, and it can sometimes be useful to discuss this confidentially, and with consent, with other senior colleagues involved in training.

When trainees are aware of my own illness, I sometimes worry that it makes it harder for them to talk of theirs. They may feel mine is ‘better’ or conversely ‘worse’, and that their illness is less important. So I try to open up generally about mental illness, in the hope that it will make it easier for them, particularly in the way that I talk about patients and other trainees.

‘Why?’ is a common question to ask, when doctors, nurses and others choose to work in psychiatry after an episode of mental illness, and there is no one answer. It is very hard to predict who will cope, who will shine, even, and who will find it hard. I can see, with hindsight, why I was advised against another long period of training and grinding exams, given the length and severity of my illness, and I now feel minimal resentment about this. I did feel angry with those who asked if I was trying to cure myself, but on the whole, they were not psychiatrists. And now, when I talk to others, I know it is important not to blind oneself with preconceptions, to know that there is not one route for all. Because I did, it does not mean all others should.

My illness has had a profound effect on my life and my work. I worked much harder and was far more organised during my psychiatric training than I had been previously. I had to be – there was always this thing at the back of my mind, this thing over which I had little control. I was determined to pass exams, even doing a Master's degree during my maternity leave. I felt that if I did not do well, my abilities would be questioned and put down to mental illness. I still think this is a difficult area; we all have times when we may perform less well, and there is little doubt that supervisors may wonder about mental health in a trainee with a history of illness, when they otherwise would not.

This is reasonable, and hopefully not punitive in any way. But as a trainee you fear assumptions, even if they are based on some truth. So supervisors must be alert, yet resist jumping to conclusions, and the way to manage this is to get to know your trainees well. It can be difficult, I know this now as a trainer; sadly, there is often nowhere where trainers and trainees all meet for coffee or lunch, the kind of things that make this happen.

### Living and surviving

It is unsurprising that I have always been interested in doctors’ accounts of mental illness. They make me feel less alone, they inspire me, and above all they are all different. The accounts by Linda Gask^23^ and Cathy Wield^24^ are moving and human, and lifted me out of my self-obsessions. They, too, are real people, things happened to them, and they are doctors. The recognition that you can recover, succeed and then get ill again was also important – these are not always stories with a happy ever after. Kay Redfield Jamison's account of having bipolar disorder is a wonderful book, describing the experience of changing moods, as well as combining this with a remarkable career researching and treating the very illness that nearly destroyed her.^25^ Mike Shooter, past president of the Royal College of Psychiatrists, describes becoming better able to recognise the warning signs of illness, and knowing when to stop.^26^

### For better or for worse

But the big question, for me, is whether my experiences make me a better doctor and psychiatrist, or even a better person. There is much written about the importance of lived experience, and it can all get a bit competitive. I trained in general adult psychiatry, but work in addiction psychiatry. I think I knew that working in general adult would potentially be harder, with reminders and triggers of what had happened to me, and that it would be very easy for me to become over-involved in a way that would be good for neither me nor patients. I am still drawn to occasional patients, where I think, ‘that could be me’, and I have to remind myself that no-one is exactly like another, no-one can experience the thoughts and suffering of someone else.

When I first started working in psychiatry, I think that I did feel that my lived experience made me better than others, and that I could more easily understand what patients were going through. I am much less sure now. There was a lightbulb moment for this, when I lost a baby late in pregnancy, early in my training. I was devastated. But it suddenly came to me that everyone's experiences are personal, that I had no idea what another mother would feel. It was a short step to realising that my experiences of altered mood, of side-effects and drugs, was only mine. I think what happened made me more alert to suffering, hopefully more empathetic and more prepared to listen. But I would never now say to someone, ‘I know exactly how you feel’.

Whether I am a better psychiatrist because of my experiences is difficult to say, as I can never know what the alternative would have been. One thing I am fairly sure of is that I am a far worse patient. I question and doubt, and my knowledge, particularly of psychiatric medication, is very unhelpful. Obviously I want to think I am a better psychiatrist, but I do not think you need to have experienced psychiatric illness to be excellent. Most of us will experience difficulty and sorrow in our lives, and these will change us and make us what we are. There is no one prescription for empathy.

## About the author

**Rebecca Lawrence** (MPhil, MSc, FRCPsych) is a Consultant Psychiatrist at Ritson Clinic in Royal Edinburgh Hospital, Scotland, UK.
